# Local approaches and ineffectivity in reducing stunting in children: A case study of policy in Indonesia

**DOI:** 10.12688/f1000research.130902.1

**Published:** 2023-02-27

**Authors:** Cashtri Meher, Fotarisman Zaluchu, Putri Chairani Eyanoer

**Affiliations:** 1 Faculty of Medicine, Universitas Islam Sumatera Utara, Medan, Indonesia; 2 Social Anthropology Department, Faculty of Social Sciences and Politics, Universitas Sumatera Utara, Medan, Indonesia; 3 Department of Community and Preventive Medicine, Faculty of Medicine, Universitas Sumatera Utara, Medan, Indonesia

**Keywords:** stunting, nutrition intervention, local context, Indonesia

## Abstract

**Background:** Stunting is a global issue. Indonesia has to cope with this stunting issue very seriously because it has the highest number among Southeast Asian countries and is included in the countries with the highest number of stunting sufferers in the world. One of the important points that requires intervention is to fulfill the nutrition of both the pregnant women and children under the age of five.

**Policy and implications:** The central government has issued the national policy to prevent stunting in children and determined the national and regional parties in charge of the program. The government commitment is shown through the fund allocation arrangement for village development aimed to prevent and overcome stunting. Theoretically, it all seemed to be conducted according to each responsibility of the parties in charge. However, the effort is generally still bureaucratic, in a form of campaign, and has not solved the issue of fulfilling nutrition at the local level.

**Recommendations:** Based on field experience and literature, the goal to reduce stunting significantly will be achievable if local approaches are applied. The local issue on nutrition intake can be intervened specifically by applying local approaches in understanding the community consumption behavior pattern and delivering education on nutrition. The government should abandon a too-generic approach (one-size-fits-all) which has been used so far.

**Conclusion:** Intervention of nutrition intake through local approaches is crucial considering that stunting prevalence is still very high. As the organization for the program to prevent stunting is supported by fund allocation, in the future the government should encourage local intervention efforts through the cooperation with higher education, local non-governmental organisations, or partners that understand local context more on nutrition issues in respective regions. To observe this, stunting prevention in Indonesia should develop more local approaches in campaign material and education to local society.

## Introduction

Stunting is a global issue. There are at least 149.2 million children in the world who are suffering from stunting. ^1^ This means those children under five years old have their height two deviation standards below the median of Child Growth Standards from the World Health Organisation (WHO). ^2^ Ironically, this global stunting prevalence does not occur evenly. The 2021 report from WHO shows that the heaviest stunting issue happens in the African and Asian areas. Those areas contribute to 82 percent of all the number of stunting cases in the world. ^1^ Meanwhile, in Southeast Asia itself, the stunting prevalence reaches 27.4 percent, ranked in the second place as the highest in all Asian areas. ^3^

As part of the areas suffering from stunting the most, the stunting data in Indonesia show a very concerning condition. Indonesia is ranked in the fifth place from the countries with the heaviest stunting problem in the world. ^4^ Even in the Southeast Asia itself, among the six countries that become the focus of malnutrition problems, Indonesia is reported to be ranked in the first place of stunting prevalence. ^5^

The high amount of stunting prevalence in Indonesia can be seen from the data of the Indonesian government. Based on the 2021 Indonesian nutritious status survey results, there are still as many as 24.7 percent of Indonesian children under the age of five suffering from stunting. ^6^ This number has shown a decrease from the stunting prevalence in 2018, which was 30.8 percent. ^7^ Nevertheless, just like the global situation picture, the stunting prevalence disparity in Indonesia is still seen very steep. From the existing 34 provinces, only six of them have under 20 percent of the stunting prevalence (with the range between 10.9 and 18.6 percent). The worst condition happens in Nusa Tenggara Timur province with the stunting prevalence as much as 37.8 percent. ^6^ Therefore, stunting in Indonesia is still a serious problem which requires better approach.

It is known that handling stunting needs to focus on the stunting issue root as the direct cause that has been known, which is lack of nutritious food intake. ^8^ The report from WHO also confirms positive results from the intervention toward the increase of food types consumed. ^9^ Therefore, nutrition intervention is the most essential thing to be conducted.

However, this food intake pattern cannot be separated from the social context existing in the community. Asian regions including Indonesia generally have certain habits, which have been believed by the community, in choosing types of food. ^10^ Moreover, the habit to manage nutrition and food is the one that contributes to the stunting problem in Indonesia. ^4^^,^^11^ In other words, local problems play an important role so that stunting prevalence in Indonesia does not experience the same burden among provinces or between areas of the same province. ^6^

Unfortunately, the stunting approach in Indonesia does not focus on the effort to further dig out this local problem. Specific intervention is still referred to as intervention only conducted by the health sector, not as the effort to dig out issues at the local level specifically. Meanwhile, sensitive intervention is also still referred to as the handling effort by the nonhealth sector. In other words, the effort to dig out local problems in giving food intake to toddlers and then to design interventions according to local context has not become the main focus from the effort to reduce stunting. As a result, Indonesian stunting reduction is still not satisfactory.

This paper will discuss the efforts that have been conducted by the Indonesian government, the implementation in the field, and the recommendations required to strengthen nutrition and food intervention more by using local approaches. We will also share our practical experiences in conducting a local approach.

## Policy outcomes and implications

Stunting does not occur in a short time, but it is a chronic condition. Stunting usually begins when a baby starts to be introduced to food; that is, when the baby is three months old until 24 months old. ^12^ Besides decreasing body immunity and increasing the possibility of being sick, stunting condition has the impact towards causing less optimal growth of children physically, mentally, and in brain capacity. ^13^^,^^14^ All of these are influential toward the capacity building of human resources (human capital), ^15^ and aggregately those influence the productivity of family, community, and even state. ^16^ The consequences toward the handling costs are very substantial. ^17^ Consequently, investments toward health of a country will also be eroded. ^18^

The Indonesian government has given full attention toward this stunting reduction. In 2021, the President of the Republic of Indonesia issued a regulation that by 2024 stunting prevalence nationally will have to be able to be reduced into just 14 percent compared to the majority of regions having over 20 percent. ^19^^,^^20^ In that regulation, the government uses a massive approach to attempt to deal with this issue. The mass approach means to involve the ministry stakeholders starting from the central to regional levels. All provinces, regencies, and municipalities in Indonesia are requested to have discussions on stunting, where each agency at every level formulates the stunting prevention program in accordance to the expertise of each agency. Every agency involving the community is also asked to deliver the campaign on the importance of preventing stunting in children. Although there is no target required, all parties are requested to reduce stunting prevalence so that it will be in accordance with the target determined by the government.

In the last ten years, the development pattern in Indonesia has had this orientation in their effort to encourage the participation of the community involved in their own village development. The government distributes the fund for village development, directly from the funds of the central government. This is carried out so that there will not be obstacles for the villages in formulating their own needs and performing the activities accordingly. This is also included in the stunting issues, and even the government determines that every village has to allocate the activities and funding from their village funds.

The Regulation of the Minister of Villages, Backward Village Development, and Transmigration Number 7/2021 states that the village fund budget can be used to prevent stunting because it is a national priority. In addition, the government keeps using stunting prevalence as the performance indicator of the Regional Heads, namely the Governors, Regents, and Mayors. ^19^^,^^21^^,^^22^

Nonetheless, the focus on the effort to discover further the problems of nutrition intake and the effort to apply local approaches is still minimally conducted. ^23^

### Implementation

In the field, the government organizes this stunting handling by implementing a dominant organization through a very bureaucratic approach. From the central government, the Vice President of the Republic of Indonesia becomes the highest rank official that leads this stunting prevention in Indonesia, and then under the Vice President there are the Vice Governors and Deputy Regents/Deputy Mayors that also lead the program in each respective region. It is expected that with the parties in charge from the central to regional governments the stunting prevention and intervention will be easily coordinated. This stunting handling coordination is conducted by holding meetings at every level, with the purpose to make sure that each role of the parties in charge is carried out. The health sector, for example, is responsible for handling stunting children cases. Meanwhile, the infrastructure development sector encourages the development of drinking water facilities and the facilities for toilets. The women empowerment sector trains the mothers to manage the health of their children. Moreover, the economic sector is expected to provide trainings to increase family income to fulfill the needs for food. The meetings are coordinated monthly, through the measurement of the children height, and it is conducted by the health workers and reported in an integrated system at the baseline data of the central government (the Ministry of Health).

Stunting indeed happens as the result of multifactor interactions directly and indirectly, ^24^ from the individual level up to the public program and policy (for instance, the assurance for food availability, market policy, and social networking). The assurance for food availability which is not sufficient in quantity and quality ^25^ along with the family status, the breastfeeding pattern, and infected diseases must indeed be organized well.

However, the involvement of the role of each stakeholder above does not seem to be integrated yet to deal with the problem of nutrition intake. The newest media report even shows that Indonesia is in the state of a very serious food starvation problem. ^26^^–^^28^ Moreover, that becomes the predictor that the real stunting problem root has not yet been dealt with.

The party that is seen to have conducted nutrition intervention may be just the health sector. Nevertheless, the health sector just applies the old approach, for instance by conducting counseling, distributing books on stunting, putting on banners, or giving biscuits produced by the Ministry of Health. All those things have been conducted as well when dealing with other diseases. Occasionally, those have to be done with limited budget, limited capability in designing a program, and the struggle to survive in the midst of the COVID-19 pandemic. In addition, a uniformed approach from the western part to the eastern part of Indonesia is again dominated by a health education programme, organized only as a regular activites. ^29^ Pursuing the target to reduce stunting causes many parties to carry out concrete actions in a form of merely giving food, but it does not solve the root of the nutrition intake problem, which should be conducted by providing knowledge to the community on the low nutrition intake and local food. The program continuity is even highly doubted because it very much depends on classical reasons, which are the government budget and personnel incapability. As a result, these mass methods then just lead to ‘unnatural’ reduction of stunting prevalence by 2024 for the sake of the government political interest, even though the real number of the decrease may not happen. This might occur considering that the stunting national survey is managed by the central government that certainly has the conflict of interest to make sure of the success mentioned in the report.

## Recommendations

Considering how little the progress has been in decreasing the stunting prevalence in the last five years in Indonesia, it is very much recommended to further evaluate the approach applied so far. Stunting handling and prevention through nutrition intervention are clearly one of the effective ways. The children under the age of five consuming food such as dairy products, vegetables, and fruits have their height improve more than those who consume grain based food or eggs, meat, poultry, and legumes. ^30^ In Guatemala, intervention by using nutritious food intake routinely, since the mothers are being pregnant, proves to be effective to reduce stunting. ^31^ Giving food in accordance with the need of the children is very necessary, ^32^^,^^33^ and this is suitable with the recommendations of WHO ^34^ on the types of food required to prevent the stunting condition.

Nonetheless, nutrition intervention really requires social studies. WHO once explained that social determinants have a crucial role toward the health status. ^35^ Nutrition social determinants can explain more or less half of the health issues. ^36^ The role of the social factor in preventing stunting has been revealed in many studies. The fact is that stunting can be ‘descended’ from a mother to her children due to the nurturing pattern and giving food tradition which will become the habit as the reference by the following generation. Therefore, it is not surprising that female empowerment and social context where women or mothers live prove to be able to improve condition. ^37^^–^^39^ This is what makes intervention impacts immensely varied, depending on the location (rural vs. urban), the geographical condition, and the local context of the local community. ^40^^,^^41^ Thus, the problem with the stunting prevalence variety which is very noticeable in countries like Indonesia should encourage the effort to dig out the causes of the low nutrition intake in a culture as well as to formulate a more specific approach to deal with it. ^42^

In other words, the too-generic approach (one-size-fits-all), which has been carried out in Indonesia so far, is not very effective and not optimal for the stunting reduction acceleration. WHO ^43^ has stated the importance to optimize the local conditions, as well as improvement among others such as the local food and local wisdom of those living and developing in the community to deal with stunting children under five and to increase the health condition of them. Moreover, the challenges to reduce stunting in the Asian region are very much related with the community habits in choosing the types of food they consume. ^10^ Despite the fact that the Asian region, including Indonesia, is a strategic location in producing agriculture, plantation, and animal husbandry products which contain proper nutritious substances, ironically it has high stunting prevalence of toddlers.

The geographical area of Indonesia is vast. There are more than 17 thousand islands with the variety of different eating pattern cultures so that this contributes to the variety of stunting prevalence. Some provinces have very high percentage of stunting prevalence, and some others have below 20 percent. Furthermore, there are some problem within a province, resulting in variations of stunting prevalence among regencies/towns. Hence, it is the local approached that are necessary to be further developed. Every region should conduct local interventions because the policy has been provided by the central government, and not tailored to each region. Every region has to deal with the stunting problem, by looking at nutrition issues in each local culture and the reasons behind the stunting. The effort to deal with stunting at the local level in order to have food intervention certainly requires strategical preliminary research, involving the community themselves, so that the intervention method can be accepted by the local people.

The local approach in designing an education module has been carried out by us before. The education display tools for the women as the training participants use local ornaments and are designed by using the local language so that they are interesting and easily learned. ^44^ The tools certainly cannot be produced without prudence in understanding local cultures. Besides that, we have designed local language poems from local songs that have been understood well by the community. ^45^ Such as this is still absent in the approaches applied so far by the Indonesian government. The food consumption context causing stunting is called as ‘the Nutritional Ecology of Stunting’ by Raiten and Bremer, (2020). ^46^ They stated that the nutrition causing stunting cannot be seen in just one perspective. There are contexts that vary and may be related to uniquely local situations.

If the local approaches and analyses are conducted, this certainly will give birth to social intervention models that are also locally focused. The local food intervention to reduce stunting has caught the attention of the practitioners in making the intervention. In the research conducted in Ethiopia, the overall stunting prevalence can be reduced significantly in two years, from 36.3 percent to 22.8 percent. The intervention applied focuses not only on the introduction of the low food diversification condition but also on the existing communication channel in the community, and then intensive collaboration among sectors can be built, including with the non-governmental organizations (NGOs). ^47^ The right communication strategy indeed should not be complicated to be carried out because the assessment toward the community local condition has been conducted. ^48^ In Bangladesh and Vietnam, the community-based model is also developed to carry out the health mobility for children under five years old. ^49^ This approach collects information involving all relevant components, among others the health workforce, parents, community elites, with the purpose to obtain not only information but also the local wisdom, as well as the communication channel that might be able to be used for the intervention phase. The comprehensive cooperation among all relevant parties, including the community, can guarantee the success of stunting prevention. ^33^ Hence, the nutrition intervention approach cannot be separated from the community socio-cultural change. It happens because food intervention has the social context suitable with the community condition.

In developing the local curriculum-based food education, the adoption of local condition has been recommended. ^50^ The project of Food, Fun, and Families (FFF) ^51^ emphasizes the importance of discovering the socio-psychological condition of the community where intervention is made, or having a project like community-based childcare centers in Malawi. ^52^ Both trial-based projects show satisfying results because since the beginning the assessment of the community condition where the intervention is made has been conducted, and that includes changing the habits of planting vegetation and managing food to be consumed by pregnant women or toddlers. The trial of Food and Agricultural Approaches to Reducing Malnutrition (FAARM) that has been conducted in Bangladesh ^53^ also focuses on food production intervention, preceded with agriculture-based socio-cultural research.

We are now using such a local approach. ^54^ Because of the mapping toward the existing issues in Nias Island, Indonesia, where the stunting prevalence is still very high, we use the local church as the main place to conduct nutrition training. The geographical location of inhabitants’ houses is far from each other, which makes it difficult to gather the people on working days. In contrast, on Sunday, the inhabitants will surely join religious activities, as this is central in the lives of the community. The role of the church is still very important so that we can see there are more than 1,000 church buildings in Nias Island for just one religious denomination. We have taken advantage of this social potential. The training method runs very effectively since all stakeholders related to nutritional intake can gather in the same place, and the mothers of the children, their husbands, their mother-in-laws, and the general community are present. Ironically, we do not see the same approach applied so far by the government, even though the provisions on the funding and intervention of stunting have been issued. Once again, the method applied so far by the government tends to be instructive only.

Therefore, the recommendations delivered above become important lessons on the global effort in accelerating stunting reduction. In this paper, the intervention practice that we have provided above is just an example from out work in the field. We cannot describe other local practices which may exist because we have so far focused on a specific location, namely Nias Island, the island located in the western part of Indonesia. However, in the future, we recommend that the government really applies the intervention practice having roots in the local community.

## Conclusion

This paper concludes that local approaches in stunting prevention in Indonesia are very much needed. The variation of the stunting problem among areas shows that local problems should not be intervened with unifomed ways. The Indonesian government needs to apply local approaches because the insignificant stunting reduction might happen due to the fact that those local approaches are ignored.

The government does not have to issue new legal rules or amend the existing rules. It just has to direct all stakeholders from the village/subdistrict level. These local approaches certainly need careful investigation. It is better that the government involves local researchers, local higher education, local NGOs so that intervention becomes more effective and has the impact toward the acceleration of stunting reduction in Indonesia.

## Data Availability

Figshare: Module about stunting for community education in Bahasa and Nias Language. https://doi.org/10.6084/m9.figshare.21938618.v2. ^44^ This project contains the following underlying data:
• Stunting bahasa Nias.pdf. (this is the module we use in teaching the community of Nias Island particularly on stunting. This module was made after conducting identification of community demography, their language, and their culture. Every page of the module contains interesting pictures, made in two languages, Indonesian language and Nias language. The language used is practical. In the module there is information on nutrition required by pregnant women and the treatment of pregnant women until they are breastfeeding their babies. The module is distributed to all the participants to be learned at home after being done with the training.) • English translation of the modul.docx. (Original text and English translation of the teaching module). Data are available under the terms of the Creative Commons Attribution 4.0 International license (CC-BY 4.0). Figshare: Song for stunting education. https://doi.org/10.6084/m9.figshare.21938624.v2. ^45^ This project contains the following underlying data:
• [REVISI] Part 2 (MV).mp4 (Educational promotional video used as part of the community outreach program. This is one of the songs whose lyric is created by us after learning about the issues in the community and is shown as part of the teaching programs. The song itself has actually long been known by the community and is often used during a wedding party. Then we created new lyrics to contain information on what can be done to prevent stunting in children. The way the song is sung is by asking one or two persons to lead the singing individually in sequence. Every time one verse is done being sung by an individual, while dancing other participants will sing the chorus part together. This song is repeatedly sung in every meeting in order to create the fun learning process and to make them remember the lyric of the song.) • English translation of the song.docx. (Original lyrics and English translation of the song). Data are available under the terms of the Creative Commons Attribution 4.0 International license (CC-BY 4.0).
