# Supplementary material for: Local approaches and ineffectivity in reducing stunting in children: A case study of policy in Indonesia
Source: F1000Res. 2023 Feb 27;12:217. [Version 1] doi: 10.12688/f1000research.130902.1 (PMC12820472; doi:10.12688/f1000research.130902.1)
Supplement: Peer review history

# Reviewer report: Reviewer response for version 1

**DOI:** 10.5256/f1000research.143699.r447612

**Published:** 2026-01-20

**Authors:** Faridah Faridah

**Affiliations:** 1 Sekolah Tinggi Ilmu Kesehatan Hang Tuah Surabaya, Surabaya, Indonesia

**Article Summary**

 This article is a policy brief that discusses the ineffectiveness of national policy approaches in reducing the prevalence of stunting in Indonesia. The author explained that despite strong political commitment, village fund allocation, and cross-sector coordination from the central to the regions, the achievement of stunting reduction is still not optimal. The root of the problem is considered to lie in an approach that is too bureaucratic, uniform (one-size-fits-all), and does not pay attention to the socio-cultural context and local food consumption patterns.

 This article combines policy analysis, literature review, and the author's field experience in conducting community-based interventions (e.g. on Nias Island). The main recommendations put forward are the need to strengthen nutrition interventions through local approaches, collaboration with universities, local NGOs, and the use of social institutions that live in the community

**Review Report**

**Question number 1**

 Answer: Partly/Partial

 Rating:

 The article quite clearly explains the context of national policies (Presidential Decree No. 72/2021, village funds, cross-sectoral roles) and the implementation structure from the central to the regions.

 The language used is relatively communicative for cross-disciplinary readers, according to the character of the policy brief.

 Concrete repair suggestions:

 Add a short subsection summarizing the national stunting policy map (actors, levels, policy instruments).

 Include a policy implementation flowchart to help non-expert readers understand the context more quickly.

 Emphasize the difference between policies on paper and implementation realities with concise, data-driven examples.

**Question number 2**

 Answer: Yes

 Minor notes:

 Some of the national policy implications (e.g. the risk of political bias in reporting stunting prevalence) are argumentative, but have not been supported by direct empirical data.

 Suggestions for improvement:

- Reinforce critical claims with policy audit data, independent evaluation reports, or cross-country comparative studies.
- Explicitly separate between literature-based findings and the author's reflective opinions.

**Question number 3**

 Answer: Yes

 Areas that can be improved:

 The recommendations are still conceptual-strategic, not equipped with success indicators or monitoring mechanisms.

**Conclusion of Scientific Assessment**

 Overall, this article is scientifically valid, relevant, and highly valuable as a policy brief in the field of public health and nutrition policy. The main strength lies in the integration of policy analysis, global literature, and local practice. However, to improve academic acumen and policy effectiveness, articles need to: 

 1. Strengthening policy analysis frameworks,

 2. Clarify the separation between data, literature, and reflective opinion,

 3. Present more operational and measurable recommendations.

Does the paper provide a comprehensive overview of the policy and the context of its implementation in a way which is accessible to a general reader?

Partly

Is the discussion on the implications clearly and accurately presented and does it cite the current literature?

Yes

Are the recommendations made clear, balanced, and justified on the basis of the presented arguments?

Yes

Reviewer Expertise:

Public health, child growth and development, social determinants of health, and analysis and implementation of health policies.

I confirm that I have read this submission and believe that I have an appropriate level of expertise to confirm that it is of an acceptable scientific standard.

# Reviewer report: Reviewer response for version 1

**DOI:** 10.5256/f1000research.143699.r447608

**Published:** 2026-01-20

**Authors:** Yuly Astuti

**Affiliations:** 1 Research Center for Population, National Research and Innovation Agency, Jakarta, Indonesia

**Policy Overview Assessment**

 The brief offers an accessible summary of national policies, statistics, and implementation (e.g., monthly meetings, sector roles like health handling cases and infrastructure building sanitation), suitable for general readers via straightforward explanations and global/Indonesian data comparisons. However, it skimps on comprehensive details like specific budgets, timelines beyond 2024 targets, or quantitative outcomes from village funds, assuming reader familiarity with disparities (e.g., 37.8% in Nusa Tenggara Timur). To enhance accessibility and soundness, authors must add a timeline table of key policies (e.g., 2018-2025 surveys) and glossaries for terms like "sensitive interventions." 

**Implications Discussion**

 Implications for human capital loss, economic costs, and policy failures (e.g., uniform biscuit distribution ignoring cultural diets) appear clearly, tied to multifactor causes like food insecurity. It accurately cites recent literature (up to 2022/2023, e.g., WHO 2021, Nutrients 2020 on nutritional ecology, Ethiopia trials reducing stunting 36.3% to 22.8%), supporting claims of social determinants explaining ~50% of issues.

**Recommendations Evaluation**

 Suggestions to prioritize local research, abandon "one-size-fits-all," and partner with local entities (e.g., using churches in Nias) are justified via authors' data-shared modules/songs and international examples (Guatemala, Malawi). They remain conceptual, lacking operational steps like funding mechanisms or metrics for "local approaches," and somewhat unbalanced by critiquing government without acknowledging partial successes (e.g., drop from 30.8% in 2018). For soundness, provide a table with phased actions:

 Phase --- Action --- Justification --- Metrics

**Phase:**

 1. Assessment

 2. Design

 3. Implementation

 4. Evaluation

**Action:**

 1. Community socio-cultural mapping

 2. Local-language tools (e.g., Nias modules)

 3. NGO/university partnerships

 4. Independent audits

**Justification:**

 1. Addresses varying provincial rates

 2. Proven in authors' work

 3. Builds on Ethiopia model

 4. Avoids conflict of interest 

**Metrics:**

 1. % households surveyed

 2. Adoption rate

 3. Stunting reduction %

 4. Pre/post prevalence 

 The table presents a phased implementation framework to ensure program soundness, transparency, and effectiveness.

**Phase 1: Assessment** focuses on *community socio-cultural mapping*. This action is essential to address wide variations in provincial outcomes by identifying local norms, beliefs, and practices that influence health behaviors. Progress at this stage is measured by the **percentage of households surveyed**, which reflects coverage and representativeness of the assessment.

**Phase 2: Design** emphasizes the development of *local-language tools*, such as context-specific modules (e.g., Nias-language materials). This approach is justified by prior empirical evidence from the authors’ work, demonstrating improved comprehension and engagement when interventions are linguistically and culturally adapted. Effectiveness is assessed through the **adoption rate** of these tools among target communities.

**Phase 3: Implementation** involves *partnerships with NGOs and universities*. This builds on successful implementation models, such as those documented in Ethiopia, where cross-institutional collaboration strengthened program delivery and sustainability. Outcomes at this phase are evaluated using the **percentage reduction in stunting**, capturing direct health impact.

**Phase 4: Evaluation** incorporates *independent audits* to ensure objectivity and accountability. This mechanism is critical to avoiding conflicts of interest and enhancing credibility of findings. Performance is measured by comparing **pre- and post-intervention prevalence rates**, allowing for rigorous assessment of program effectiveness over time.

 Furthermore, to achieve full approval (post-reservations), address these must-fix points:

- **Clarify methodology:** Explicitly state as "reflective policy analysis with case study" to position beyond empirical study; detail Nias data collection (sample size, methods).
- **Operationalize concepts:** Define "local approaches" (e.g., "interventions incorporating ≥3 cultural elements like language/venues") and "ineffectivity" (e.g., <5% annual decline despite funding).
- **Balance critique:** Quantify successes (e.g., post-2021 progress) and risks of decentralization (e.g., capacity gaps).
- **Update literature:** Add 2024-2026 stunting data if available; ensure all claims (e.g., food crises) link to sources.
  These changes, without new data, would elevate rigor while retaining policy relevance.

Does the paper provide a comprehensive overview of the policy and the context of its implementation in a way which is accessible to a general reader?

Partly

Is the discussion on the implications clearly and accurately presented and does it cite the current literature?

Yes

Are the recommendations made clear, balanced, and justified on the basis of the presented arguments?

Partly

Reviewer Expertise:

My research expertise lies in population health, with a strong focus on traditional practices related to health behavior and broader social factors influencing health outcomes.

I confirm that I have read this submission and believe that I have an appropriate level of expertise to confirm that it is of an acceptable scientific standard, however I have significant reservations, as outlined above.

# Reviewer report: Reviewer response for version 1

**DOI:** 10.5256/f1000research.143699.r437796

**Published:** 2025-12-31

**Authors:** Fatria Harwanto

**Affiliations:** 1 Sriwijaya University, Palembang and Indralaya, Indonesia

This policy brief examines the factors underlying the limited effectiveness of stunting reduction efforts in Indonesia, despite strong political commitment, substantial financial investment, and extensive multi-sectoral coordination. The authors argue that the principal weakness of current policies lies in the reliance on bureaucratic, generic, and centrally designed interventions, which insufficiently account for local socio cultural determinants of nutrition and dietary practices.

 Drawing on policy analysis, a review of the relevant literature, and the authors’ field-based experience sparticularly in Nias Island, the paper demonstrates that locally tailored nutrition education and intervention strategies, which incorporate local languages, community institutions, and culturally embedded practices, are likely to be more effective than uniform, nationwide campaigns. The article concludes that stunting prevention in Indonesia requires a fundamental paradigm shift, moving away from standardized, top down approaches toward context-sensitive, community based, and participatory strategies.

 This article demonstrates strong policy relevance, coherent argumentation, and adequate support from the existing literature, particularly in highlighting the importance of local approaches in stunting prevention in Indonesia. However, in its current version, the article does not yet fully meet academic standards, particularly with regard to: The lack of clarity in its methodological positioning (i.e., whether it is intended as a policy analysis or a reflective policy paper rather than an empirical study),The insufficient operationalization of key concepts, such as local approaches and policy ineffectiveness, Recommendations that remain largely conceptual and would benefit from being articulated in a more operational and actionable manner. These limitations can be addressed through clear and targeted revisions, without the need for additional data collection or substantial changes to the core focus of the article

Does the paper provide a comprehensive overview of the policy and the context of its implementation in a way which is accessible to a general reader?

Partly

Is the discussion on the implications clearly and accurately presented and does it cite the current literature?

Yes

Are the recommendations made clear, balanced, and justified on the basis of the presented arguments?

Partly

Reviewer Expertise:

NA

I confirm that I have read this submission and believe that I have an appropriate level of expertise to confirm that it is of an acceptable scientific standard, however I have significant reservations, as outlined above.
